# Benign pericardial schwannoma in a Chinese woman: a case report

**DOI:** 10.1186/1471-2261-13-45

**Published:** 2013-06-24

**Authors:** Xu-Hui Zhang, Yu Wang, Xian-Yue Quan, Bo Liang

**Affiliations:** 1 Department of Radiology, Zhujiang Hospital, Southern Medical University, Guangzhou 510282, China; 2 Department of Pathology, Zhujiang Hospital, Southern Medical University, Guangzhou 510282, China

**Keywords:** Pericardium, Benign schwannoma, Computed tomography

## Abstract

**Background:**

Intrathoracic schwannomas are most frequently located in the posterior mediastinum. A Chinese woman presented with a benign pericardial schwannoma in the pretracheal space and aortopulmonary window, a location which has not been described previously in the literature.

**Case presentation:**

A 50-year-old Chinese woman initially reported a cough associated with a small amount of sputum. Contrast-enhanced computed tomography (CT) subsequently revealed a 9 × 11 cm^2^ lobulated mass with sharp margins that presented as a capsule with heterogeneous enhancement and punctate calcification. Complete surgical resection was performed using a thoracotomy approach. The resected intrapericardial tumor was a firm, large mass with lobulation. Capsulation prevented infiltration of the mass into adjacent organs. Pathological examination verified that the tumor was a benign pericardial schwannoma.

**Conclusion:**

This is the first reported case of a benign pericardial schwannoma located in the pretracheal space and aortopulmonary window. While a contrast-enhanced CT scan was able to differentiate this pericardial schwannoma from other middle mediastinal tumors, the exact diagnosis and plan for treatment depended on a pathological examination. For similar cases involving pericardial schwannomas, complete surgical resection is recommended, particularly for the prevention of life-threatening cardiopulmonary complications.

## Background

Schwannomas are benign, slow-growing, epineurium-encapsulated neoplasms arising from Schwann cells and may develop in any part of the body [1,2]. Intrathoracic Schwannomas are most frequently located in the posterior mediastinum. Primary schwannomas arising from the pericardium are extremely rare. To our knowledge, a case involving a malignant schwannoma distributed over the heart was reported only recently by Amato *et* al. [3]. In that case, this schwannoma had invaded the pericardium. In the present report, a novel finding of a Chinese woman with a benign pericardial schwannoma located in the pretracheal space and aortopulmonary window is described.

## Case presentation

A 50-year-old Chinese woman reported the presence of a cough associated with a small amount of sputum for the previous month, and a fever over the previous week. A physical examination and laboratorytests showed no abnormal findings.

**Figure 1:**
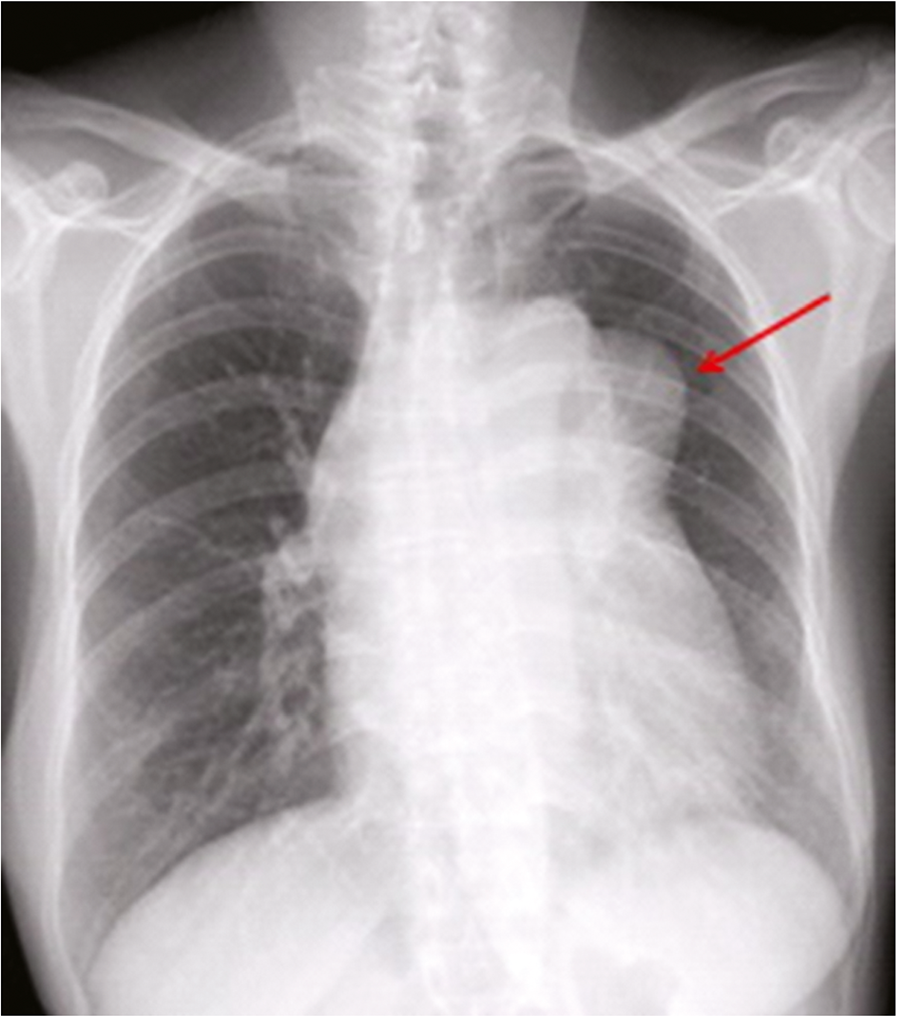
Posteroanterior radiograph showing the mass of interest (indicated with a long arrow) protruding from the pulmonary segment.

**Figure 2:**
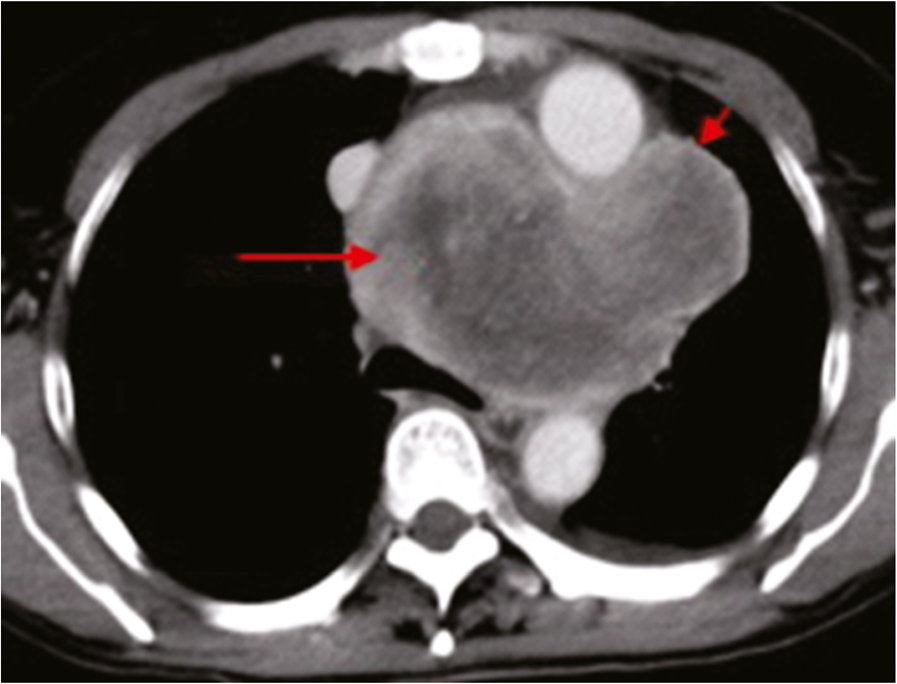
**Contrast-enhanced CT detected a large, wel-defined, lobulated mass (indicated with long arrows) associated with heterogeneous enhancement in the pretracheal space and aortopulmonary window.** The capsule of the mass (indicated with short arrows) also exhibited enhancement.

When a posteroanterior chest radiograph was obtained, a widened mediastinum, a mass protruding from the pulmonary segment were detected (Figure 1). Using contrast-enhanced computed tomography (CT), a 9 × 11 cm^2^ lobulated mass exhibiting heterogeneous enhancement and punctate calcification with sharp margins was observed. The capsule of the mass was enhanced in the CT images. The mass was located in the pretracheal space and aortopulmonary window (Figure 2), directly adjacent to, and displacing, the superior vena cava (SVC), ascending and descending aorta, pulmonary artery, left atrium, trachea, and primary bronchi. In addition, small amounts of pericardial and left pleural effusion were observed (Figure 2).

**Figure 3:**
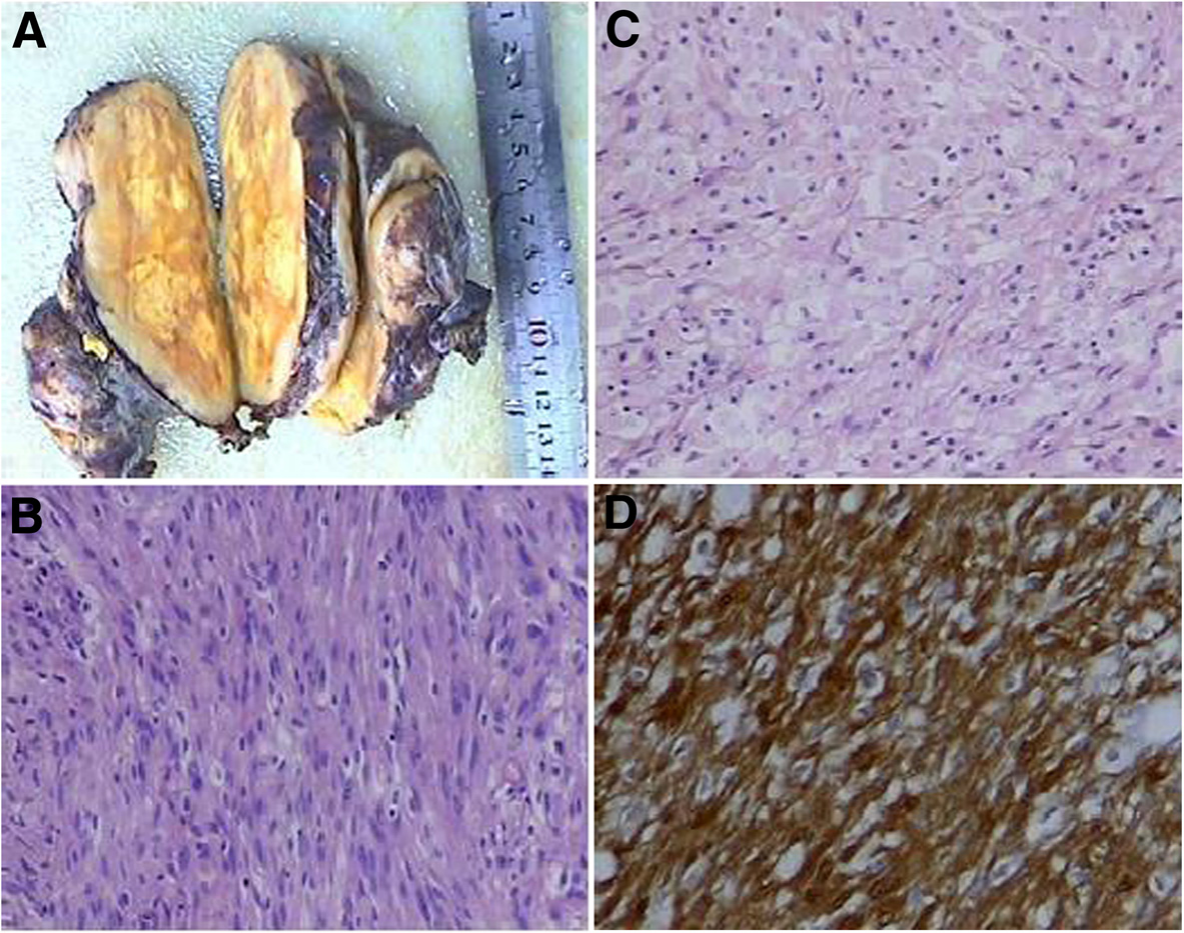
**Histopathological findings. A**) Examination of the resected, encapsulated tumor specimen revealed a grey-yellow colored surface, and a grey-white colored surrounding region. **B**) Antoni A area composed of ill-defined fascicles of spindle cells (Magnification, 100×). **C**) Loosely organized Antoni B area (magnification, 100×). **D**) Strongly positive immunohistochemical staining for S-100 protein (magnification, 200×).

Complete surgical resection was performed via a thoracotomy approach. The resected intrapericardial tumor was large in size and firm. The tumor was situated under the ascending aorta, with its right edge adhered to SVC, and was compressing the SVC and right atrium. The upper edge of the tumor was adjacent to the aortic arch, the left edge was attached to the pericardium and left hilus of lung, the lower edge was adhered to left atrium and pulmonary artery, and the posterior edge was adjacent to the trachea, primary bronchi and the descending thoracic aorta, The tumor compressed the trachea and was pushing the primary bronchi toward the vertebral column. The tumor exhibited lobulation and capsulation (Figure 3A). Correspondingly, no infiltration of adjacent organs was observed.

Histological examination revealed that the tumor was made up of two tissue types: Antoni A and Antoni B. The Antoni A type tissue was composed of spindle cells that were closely packed together and arranged in bundles and rows with palisading nuclei without mitoses (Figure 3B). The Antoni B type tissue had a low density of cells that were dispersed in a loose and random fashion; the tumor cells were polygonal with abundant cytoplasm, cytoplasmic lipids, and had round or oval nuclei (Figure 3C). The tumor cells were strongly immuno positive for S-100 protein (Figure 3D).Based on these results, a diagnosis of benign pericardial schwannoma was made.

The patient’s general health was good. She exhibited no evidence of recurrence at a follow-up CT scan performed 5 months after the surgical resection.

## Conclusions

Primary schwannomas arising from the pericardium are extremely rare. Therefore, the present case of a benign pericardial schwannoma located in the pretracheal space and aortopulmonary window is of great interest. Moreover, to our knowledge, this case represents the first of its type to be reported.

Since chest radiography does not establish a specific diagnosis of pericardial schwannoma, CT scans are used for precise localization and determination of the tumor’s approximate size and internal characteristics, as well as its proximity to surrounding structures [4,5]. In the present case, a pericardial schwannoma was detected in the pretracheal space and aortopulmonary window in a CT scan. This tumor location has not been reported previously for pericardial schwannomas. The schwannoma had a capsule and exhibited heterogeneous enhancement and punctate calcification. These characteristics are consistent with those previously described for schwannomas [4]. Several reports have also suggested that a fibrous capsule is a key feature of schwannomas and may represent a characteristic that facilitates differential diagnoses of schwannomas from other pericardial tumors in imaging studies [4-6].

The benign pericardial schwannoma should be differentially diagnosed from other tumors that occur in the pretracheal space and aortopulmonary window, such as bronchial cysts, lymphomas, and metastatic lymphadenectasis. Bronchogenic cysts appear as single, smooth, round or elliptic non-enhanced masses with homogeneous attention. Lymphomas can manifest as multiple, rounded, and conglomerate soft-tissue masses with the capacity to infiltrate or invade neighboring vascular structures. Metastatic lymph nodes have solitary or multiple round soft tissue shadows in CT scans, can be integrated into clusters, and may be oppressing or infiltrating adjacent organs. Necrosis sometimes occurs in the nodes and patients with metastatic lymph nodes often have a history of malignant tumors.

In most tumor cases, the exact diagnosis and plan for treatment are dependent upon pathological findings. Microscopically, the tumor in our patient was consistent with the typical schwannoma composition, including both hypercellular Antoni A and hypocellular Antoni B areas. Likewise, immunohistochemically, the tumor in our patient was strongly positive for S-100 protein, a schwannoma marker [7-9], and the tumor cells did not have mitotic figures. Furthermore, the tumor had not infiltrated adjacent structures. Based on these observations, the tumor was diagnosed as a benign pericardial schwannoma, and complete surgical resection was successfully performed via a thoracotomy approach. Following the operation, the patient experienced an uneventful recovery.

In conclusion, this is the first reported case of a benign pericardial schwannoma located in the pretracheal space and aortopulmonary window. Although a contrast-enhanced CT scan enabled us to differentiate this pericardial schwannoma from other middle mediastinal tumors, the exact diagnosis and plan for treatment depended on the pathological findings. For similar cases involving pericardial schwannomas, complete surgical resection is recommended, particularly for the prevention of life-threatening cardiopulmonary complications.

## Consent

Written informed consent was obtained from the patient for publication of this case report and the accompanying images. A copy of the written consent form is available for review by the Editor of this journal.

## Abbreviations

CT: Computed tomography; SVC: Superior vena cava.

## Competing interests

The authors declare that they have no competing interests.

## Authors’ contributions

ZXH performed data analyses and wrote the manuscript. ZXH, WY, QXY, and LB conducted the clinical diagnosis and data collection. All authors read and approved the final manuscript.

## Pre-publication history

The pre-publication history for this paper can be accessed here:

http://www.biomedcentral.com/1471-2261/13/45/prepub
